# The Addition of Extra-Virgin Olive Oil Enhances the Antioxidant Capacity, Nutritional Quality, and Sensory Attributes of Vegetable Patties Prepared Using Different Cooking Methods

**DOI:** 10.3390/foods15020366

**Published:** 2026-01-20

**Authors:** Andrés Bustamante, Consuelo Valle, Francisca Echeverría, Elva Gonzales, Mónica Cabrales, Camila Farias, Yasna Muñoz, Beatriz Reyes, Lorena Mercado-López, Rodrigo Valenzuela

**Affiliations:** 1 Department of Nutrition, Faculty of Medicine, University of Chile, Santiago 8350499, Chile; anbustama@uchile.cl (A.B.); elvagnutricion@gmail.com (E.G.); camila.farias.c@uchile.cl (C.F.); yasnamunoz@uchile.cl (Y.M.); breyesp13@gmail.com (B.R.); lorena.mercado@gmail.com (L.M.-L.); 2 Departamento de Nutrición y Dietética, Escuela de Ciencias de la Salud, Facultad de Medicina, Pontificia Universidad Católica de Chile, Santiago 8331150, Chile; franciscaecheverria@uc.cl; 3 Facultad de Farmacia, Escuela de Nutrición y Dietética, Universidad de Valparaíso, Valparaíso 2360102, Chile; 4 Facultad de Salud, Escuela de Nutrición y Dietética, Universidad Santo Tomás, Los Ángeles 4441171, Chile; 5 Departamento de Postgrado, Facultad de Medicina, Universidad Andrés Bello, Viña del Mar 8370035, Chile

**Keywords:** plant-based foods, fatty acid composition, monounsaturated fatty acids, polyphenols, frying

## Abstract

The growing demand for healthier and more sustainable foods has driven interest in plant-based formulations with improved nutritional and sensory quality. In this context, this study evaluated the effect of extra-virgin olive oil (EVOO) addition and different cooking methods (baking, air-frying, and deep-frying) on the nutritional composition, fatty acid profile, total polyphenol content (TPC), antioxidant capacity (ORAC, FRAP, and DPPH), and sensory acceptability of vegetable patties. Patties were prepared with or without EVOO and cooked using the three methods. Deep-frying markedly increased fat content (45–75%), whereas baking and air-frying effectively limited oil absorption (0–10%). EVOO addition increased monounsaturated fatty acids, particularly oleic acid (37.2 g/100 g DW), and enhanced the transfer of phenolic compounds to the patties. Deep-fried, EVOO-enriched samples showed the highest TPC (3.93–5.22 mg GAE/100 g DW), while raw patties exhibited the highest ORAC values (5.17–6.02 µmol TE/100 g DW). Sensory evaluation indicated that EVOO-enriched patties achieved the highest overall preference when air-fried or baked (77–89%). Overall, enriching EVOO with less oil-intensive cooking methods improved the lipid profile, antioxidant capacity, and sensory quality of vegetable patties. These findings should be interpreted within the context of a single frying cycle representative of domestic cooking practices and a sensory evaluation conducted with an untrained panel mainly composed of young adults.

## 1. Introduction

In 2019, a transformation towards healthier and more sustainable diets by 2050 was proposed, benefiting both human health and the planet [1]. A flexitarian approach was suggested, emphasizing plant-based foods such as fruits, vegetables, nuts, legumes, and whole grains, with moderate amounts of meat and dairy, and a 50% reduction in red meat and sugar intake [2]. This model encompasses dietary patterns ranging from the Mediterranean to vegan and vegetarian diets, characterized by a high content of plant-based foods and low intake of animal-derived products [3,4,5]. In response, the food industry has developed products with higher vegetable content and high-quality protein sources, contributing to a healthier and more sustainable food supply [6]. Within this context, plant-based patties have gained attention as meat alternatives that replicate the sensory attributes of traditional burgers while partially or entirely excluding animal-derived ingredients [7,8]. Their rapid market expansion reflects growing consumer demand for sustainable protein sources, although their nutritional and sensory profiles vary considerably among products [9]. In addition to formulation, the cooking method significantly influences the sensory characteristics and chemical composition of patties [10].

One of the most traditional cooking methods is deep-frying, in which foods are immersed in hot oil (160–180 °C) [11]. When food is introduced into the oil, surface water reaches its boiling point, triggering heat transfer from the oil to the food’s surface and interior. This process forms a crust and induces physical and chemical transformations, including cell wall rupture, pore formation, core temperature increase, moisture loss, starch gelatinization, and protein denaturation, resulting in a crispy, golden exterior [12]. Recently, air-frying has emerged as a healthier alternative, using rapid hot-air circulation instead of immersion in oil. In a comparative study on falafel, Fikry et al. reported that air-frying at optimal temperature and time preserved sensory properties while reducing fat content by up to 70% compared to deep-frying. Such technologies offer promising options for producing foods with lower fat and calorie content without compromising taste and texture [13].

During deep-frying, the choice of oil is a key factor in obtaining a healthier product. Extra-virgin olive oil (EVOO), a central component of the Mediterranean diet, is notable for its favorable fatty acid profile, with high monounsaturated fatty acids (MUFA; oleic acid (OA) C18:1 n-9, 65–72%), moderate saturated fatty acids (SFA; palmitic acid C16:0, 12–16%), and lower polyunsaturated fatty acids (PUFA; linoleic acid (LA) C18:2 n-6, 10.61–18.33%) [14]. EVOO is also rich in phenolic compounds (e.g., oleuropein, tyrosol, hydroxytyrosol) and contains tocopherols (α, β, γ, δ) with antioxidant properties [15]. This antioxidant richness helps reduce oxidation during prolonged cooking [16,17]. Moreover, EVOO can transfer bioactive compounds to foods during frying, thereby improving their nutritional profile [18]. However, some antioxidants may remain in the oil, especially when subjected to high temperatures or prolonged cooking [19]. Alternative cooking methods, such as baking, air-frying, or grilling, may enhance the phenolic compound content through dehydration, facilitating their release from the food matrix [20]. Despite these benefits, little is known about the antioxidant capacity of EVOO when used to cook vegetable-based patties with different techniques. In this context, developing a vegetable-based patty with EVOO could optimize nutritional and functional properties by combining the benefits of MUFA and antioxidants from the oil. Therefore, this study aims to formulate and assess the nutritional profile and quality of vegetable-based patties with EVOO cooked by various techniques.

## 2. Materials and Methods

### 2.1. Chemicals and Reagents

All chemicals and reagents used in the analyses were of analytical or chromatographic grade. Chloroform, methanol, hexane, sodium hydroxide (NaOH), boron trifluoride in methanol (BF_3_–methanol, 20%), sodium chloride (NaCl), sodium carbonate (Na_2_CO_3_), potassium chloride (KCl), disodium hydrogen phosphate (Na_2_HPO_4_), potassium dihydrogen phosphate (KH_2_PO_4_), acetate buffer reagents, hydrochloric acid (HCl), sulfuric acid (H_2_SO_4_), copper sulfate (CuSO_4_), potassium sulfate (K_2_SO_4_), ammonia solution (NH_3_), phenolphthalein, methylene red indicator, ferric chloride hexahydrate (FeCl_3_·6H_2_O), and butylated hydroxytoluene (BHT) were purchased from Merck, Darmstadt, Germany. Sodium hydroxide (0.5 N), sulfuric acid (0.1 N), and other standardized solutions were prepared according to AOAC procedures. The internal standard tricosanoic acid methyl ester (C23:0) was obtained from Nu-Check Prep, Elysian, MN, USA. Folin–Ciocalteu reagent, gallic acid, fluorescein, 2,2-azobis(2-amidinopropane) dihydrochloride (AAPH), 2,2-diphenyl-1-picrylhydrazyl (DPPH), 2,4,6-tripyridyl-s-triazine (TPTZ), ferric chloride, and 6-hydroxy-2,5,7,8-tetramethylchroman-2-carboxylic acid (Trolox) were supplied by Sigma–Aldrich, St. Louis, MO, USA. Distilled and deionized water were used throughout the analyses, depending on the specific analytical procedure.

### 2.2. Vegetable Patties Formulation and Cooking

**Figure 1:**
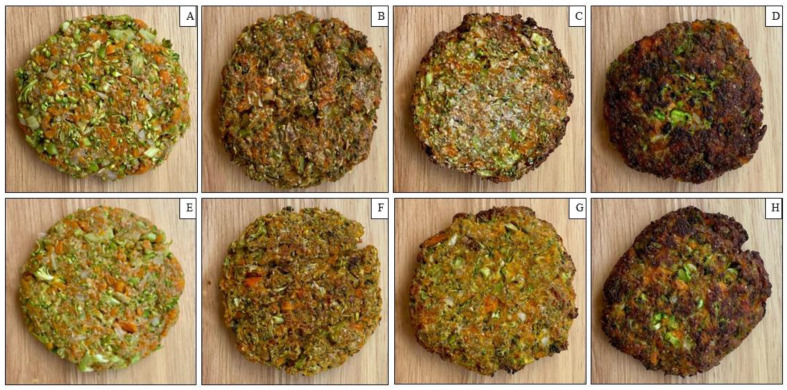
Representative images of vegetable patties with and without EVOO using different cooking methods. (**A**) Raw EVOO (−); (**B**) Baked EVOO (−); (**C**) Air-fried EVOO (−); (**D**) Deep-fried EVOO (−); (**E**) Raw EVOO (+); (**F**) Baked EVOO (+); (**G**) Air-fried EVOO (+); (**H**) Deep-fried EVOO (+).

The patties were prepared with finely chopped cooked broccoli (35%), mashed carrots (28%), finely chopped onion (9%), egg white (10.3%), oat flour (17%), salt (0.5%), pepper (0.1%), garlic powder (0.1%), and cumin (0.1%). A total of 42 patties were produced, half of which were enriched with 2.5 mL (4.5%) of EVOO during preparation (*n* = 21 with EVOO and *n* = 21 without EVOO). The EVOO used was of the Arbequina cultivar (Santa Elvira brand, Cauquenes, Maule Region, Chile), with a free acidity of less than 0.2% and a total polyphenol content of 22 mg gallic acid equivalents (GAE) per 100 g of oil. The patties (with and without EVOO) were divided into three groups (*n* = 7 units each) and subjected to different cooking methods: (1) baking (control); (2) deep-frying; (3) air-frying (Figure 1). The procedures were as follows: baking was performed in an oven (Electrolux air-o-steam Touchline 10 GN 2/1, Stockholm, Sweden) at 180 °C for 15 min; air-frying was carried out in an air fryer (Fry Delight Tefal, Shaoxing, China) at 180 °C for the same duration; and deep-frying was conducted in a fryer (Somela Deep Fryer DF535T, Santiago, Chile) at 170 °C for 2 min. All experiments were performed in triplicate.

### 2.3. Proximate Chemical Analysis

A chemical analysis of the raw and cooked samples was conducted to quantify moisture, ash, protein, and total fat, following methods established by the Association of Official Analytical Chemists (AOAC). Moisture was measured using a moisture analyzer (PMR-50, Radwag, Radom, Poland), where the sample was placed in a metallic capsule and dried for 60 to 90 min (AOAC Method 942.15). Ash content was analyzed using a muffle furnace (Thermolyne Type 6000 Furnace, Thermolyne, Cajon, CA, USA) through the AOAC Method 940.26, by incinerating 5 g of the dehydrated sample at 525 °C for approximately 7 h. Protein content was determined via the Kjeldahl method (AOAC 981.10) using 1.5 g of patty sample. Fat content was determined using the Bligh & Dyer method (AOAC 923.05) [21]. Approximately 5 g of sample with 80% moisture was homogenized with 200 μL of BHT, 5 mL of chloroform, and 10 mL of methanol using a homogenizer (SHM1, Stuart). Phase separation was achieved by the addition of chloroform and distilled water, and the lipid fraction was recovered. Solvents were removed under reduced pressure using a rotary evaporator (R-100, BÜCHI, Geneva, Switzerland) at 37 °C, followed by oven drying at 105 °C (UF-260, Memmert, Schwabach, Germany) to constant weight. Carbohydrates were calculated by differences, subtracting the percentages of moisture, ash, protein, and total fat. Water activity (A_W_) was also determined using an HD-6 water activity meter (MRC) by filling a plastic capsule three-quarters of its volume and measuring the value after 20 min [21]. The results were expressed on a dry basis as mean values ± standard deviation.

### 2.4. Fatty Acid Composition

The fatty acid composition was determined in the total lipid fraction previously extracted from cooked patties using the Bligh and Dyer method [21], as well as in the oil used for deep-frying. Fatty acid methyl esters (FAMEs) were prepared following the procedure described by Morrison and Smith et al. [22]. For the analysis, 200 µL of oil sample was placed in a test tube along with 1 mL of C23:0 as an internal standard and 0.5 mL of a NaOH solution (0.5 N) in methanol. The mixture was vigorously vortexed and heated in a water bath at 90–95 °C for 10 min. After heating, the mixture was cooled, and 1 mL of BF3 (20% methanol) was added. The heating process was then repeated in the water bath for an additional 10 min. After cooling again, 3 mL of saturated NaCl and 1 mL of hexane were added, followed by vortexing to extract the upper phase. The extracted lipids were converted into FAMEs, which were analyzed by gas chromatography (GC) using an Agilent 7890A (Agilent Technologies, Santa Clara, CA, USA) system equipped with an Agilent HP-88 capillary column (60 m × 0.25 mm; ID 0.25 mm) and a flame ionization detector. The internal standard C23:0 was used to calibrate the results, which were expressed as grams of fatty acids per 100 g of patty sample or oil.

### 2.5. Total Polyphenol Content and Antioxidant Capacity

#### 2.5.1. Extraction Process

The extraction of antioxidants from the samples was performed using the method described by Montedoro et al. [23]. For this, 5 g of the sample was mixed with 30 mL of 80% methanol and agitated in an orbital shaker (JSR Cientec, Santo Domingo, Dominican Republic) at 150 RPM for 2 h. The resulting mixture was transferred to a Falcon tube and centrifuged (Allegra 21R Centrifuge, Beckman, Pasadena, CA, USA) at 4000 rpm for 5 min. The supernatant was recovered, and the final volume was adjusted to 25 mL. To extract antioxidants from the oil, 3 mL of oil was mixed with 3 mL of 80% methanol, vortexed for 15 s, and sonicated for 15 min. The mixture was then centrifuged (Allegra 21R Centrifuge, Beckman) at 4000 rpm for 5 min, and the supernatant was transferred to a 10 mL volumetric flask. This process was repeated three times, and the volume was completed with 80% methanol.

#### 2.5.2. Total Polyphenol Content

The total polyphenol content (TPC) was determined using the Folin–Ciocalteu method, as described in the protocol of Singleton et al. [24]. In a 10 mL test tube, 0.1 mL of the extracted antioxidant sample was mixed with 4.9 mL of distilled water and 0.5 mL of the Folin–Ciocalteu reagent. After 3 min, 1.7 mL of a 20% sodium carbonate solution was added, and the volume was completed with distilled water. The tube was sealed, agitated, and left to rest for 30 min. Absorbance was measured at 765 nm using a spectrophotometer (Lambda 25, PerkinElmer, Singapore). The results were expressed as mg of GAE per 100 g of patty on a dry basis ± standard deviation.

#### 2.5.3. Antioxidant Capacity

The antioxidant capacity was assessed by the Oxygen Radical Absorbance Capacity (ORAC), Ferric Reducing Antioxidant Power (FRAP), DPPH methods. Briefly, the ORAC assay was performed according to the method of Abreu et al. [25] using black 96-well polystyrene microplates. A calibration curve was prepared with concentrations of 12.5 μM, 25.0 μM, 50.0 μM, and 100.0 μM. In the wells, 25 μL of the antioxidant samples were added, with each sample replicated six times, and readings were performed using the Synergy HT Biotek Gen 5 2.09 equipment. Fluorescein and an AAPH solution were added to each well, and the area under the fluorescence curve (AUC) was measured. The results were expressed as the mmol of Trolox equivalents (TE) per 100 g dry weight patty ± standard deviation. FRAP assay followed the method of Benzie and Strain et al. [26]. Absorbance was measured at 593 nm using a spectrophotometer (Lambda 25, PerkinElmer Singapore). The results were expressed as mmol of TE per 100 g dry weight patty ± standard deviation. Finally, DPPH analysis was conducted using the method of Brand-Williams et al. [27]. Absorbance was measured again at 517 nm using a spectrophotometer (Lambda 25, PerkinElmer Singapore). The results are reported as mmol of TE per 100 g dry weight patty ± standard deviation. All analyses were performed in triplicate.

### 2.6. Sensory Evaluation

The sensory evaluation was conducted with untrained consumers in accordance with the guidelines of ISO 11136:2014 [28]. A total of 53 participants were recruited, tasting vegetable patties with and without EVOO, subjected to baking, air-frying, and deep-frying with EVOO. Each sample, along with its respective cooking method, was assigned three random numbers and presented on plates in a standardized manner, following a random, pre-established order. After reading and signing the informed consent form (Project No. 003-2023, Minutes File No. 037), participants were provided with the corresponding evaluation form, along with the samples in the assigned order and a glass of water. This process was repeated for each cooking method. The evaluation included: (1) Consumer profile: Participants indicated their age range and gender. (2) Consumption filter: They marked products they do not consume or avoid and declared any food allergies (list: broccoli, carrot, egg, onion, pepper, and oatmeal). (3) Acceptability test: Participants evaluated the samples using a 7 point hedonic scale ranging from 1: “dislike very much” to 7: “like very much” sensory for attributes such as appearance, aroma, taste and texture. (4) Overall preference was assessed by asking consumers to indicate their preferred sample after sensory evaluation.

### 2.7. Sample Size Calculation and Statistical Analysis

Pattie’s sample size was calculated using an Excel template specifically designed for comparing two means. The study by De Alzaa et al. [18], which reported a standard deviation of 0.06 in the fatty acid composition of EVOO during the deep-frying of various products, was used as a reference. A 95% confidence interval and 90% statistical power were applied, along with a margin of error of 0.1, resulting in a sample size of 6 units per group. To account for potential losses, an additional 15% of samples were included, yielding 7 units per group per cooking method. For the sensory panel, more than 50 untrained participants will be included, in accordance with ISO 11136:2014, ensuring an adequate statistical power to detect significant differences in the responses. Statistical analyses were performed to determine significant differences in the results of proximal chemical analyses, fatty acid composition, antioxidant capacity, and sensory evaluation, considering EVOO enrichment (−/+) and cooking method as the independent factors. A two-way analysis of variance (ANOVA) followed by Tukey’s HSD multiple comparison test, with a significance level of 95% (*p* < 0.05), was conducted using GraphPad Prism 10.6.0.

Additionally, multivariate analysis was conducted using Principal Component Analysis (PCA) and correlogram analysis to investigate the relationships among variables and sample clustering. Prior to PCA, data were standardized. PCA and correlograms were conducted using RStudio (version 2025.09.2+418) and R software (version 4.4.3). 

## 3. Results and Discussion

### 3.1. Proximate Composition and Fatty Acid Profile of the Patties and Deep-Frying Oil

The selected time-temperature combinations in this work were based on achieving adequate cooking of the vegetable patties under each method, rather than ensuring equivalent thermal exposure across treatments. In this study, baking and air-frying were performed at 180 °C for 15 min, whereas deep-frying was performed at 170 °C for 2 min. Deep-frying is a high-temperature, short-time process in which immersion in hot oil enables rapid heat transfer, promoting accelerated moisture evaporation and structural changes in the food [29]. In contrast, air-frying and oven baking rely on hot air as the heating medium, which requires longer processing times to reach comparable levels of cooking and internal doneness [30]. Accordingly, these different cooking conditions reflect the intrinsic heat transfer mechanisms of each method and provide the basis for interpreting the observed changes in proximate composition, fatty acid profile, phenolic content, antioxidant capacity, and sensory attributes discussed below.

The proximate composition of vegetable patties with and without EVOO is shown in Table 1. Regarding moisture content, a significant decrease was observed in both EVOO-enriched and non-enriched vegetable patties after they were deep-fried (17–18%). In line with these results, water activity (A_W_) was significantly lower in raw patties, both with and without EVOO, compared to deep-fried samples. Similarly, the protein content also showed a significant reduction in both types of patties when subjected to frying (27.5–29.5%). As expected, the fat content increased markedly in all patties, regardless of EVOO addition, when they were deep-fried compared to other cooking methods (45% to 75%). Additionally, EVOO-enriched patties showed significant differences in fat content when baked, whereas non-enriched patties exhibited no significant variation between baking and air-frying (Table 1). When comparing samples across cooking methods, non-enriched patties subjected to deep-frying, as well as EVOO-enriched patties in both raw and cooked forms, exhibited significant differences in moisture, protein, fat, and total carbohydrate compared to other samples.

Numerous studies have demonstrated that deep-frying significantly increases the fat content of foods, influenced primarily by the food matrix and its initial moisture content [12,31,32,33]. Alkaltham et al. [31]. reported a notable increase in oil absorption in pretreated fried potatoes, where blanching significantly enhanced this effect after frying at 180 °C for 3 min. In the present study, patties with higher initial moisture content also showed greater oil absorption, likely due to the cooking process. Specifically, boiling carrots as an initial preparation step before blending with other ingredients increased the moisture content of the raw patties [11,34].

All cooking methods (baking, air-frying, and deep-frying) significantly reduced the patties’ moisture content, with an average reduction of 60%, in agreement with previous studies [35,36]. During deep-frying, water evaporation generates steam pressure, which degrades the food’s structure and facilitates oil penetration, resulting in higher oil uptake [37,38]. This aligns with previous studies [39,40], as total fat content in the present study increased substantially (by 45–75%) with deep-frying compared to air-frying and baking. This increase was directly correlated with the patties’ initial moisture content (22.3–32.11 g/100 g dry weight). In contrast, air-frying has proven to be an effective alternative for reducing fat content, improving the nutritional profile, and offering health benefits, as well as ecological advantages by minimizing oil use and eliminating effluents associated with deep-frying [41,42].

The higher A_W_ values observed in deep-fried patties could reflect localized high-moisture zones resulting from crust formation, internal condensation phenomena, and matrix reorganization. Although deep-frying leads to substantial surface moisture loss, it does not necessarily result in a uniform reduction in A_W_ throughout the product. Rapid dehydration of the outer layers promotes crust formation, which acts as a barrier to further moisture loss, while the inner regions remain humid [43]. Previous modeling and experimental studies have demonstrated that water evaporated from the crust can diffuse and condense within the porous interior, resulting in heterogeneous moisture distribution and localized regions with higher A_W_, despite an overall decrease in total moisture [44]. In addition, thermal-induced matrix changes, including protein denaturation and starch gelatinization, modify water-binding and water-holding properties, potentially increasing the proportion of free water within the food matrix [45].

Regarding protein content, both EVOO-enriched and non-enriched patties subjected to deep-frying showed significantly lower protein values compared to those prepared using other cooking methods. This reduction is attributed to protein denaturation, a characteristic phenomenon of deep-frying [46]. The egg white included in the formulation, rich in water and albumin, undergoes ovalbumin degradation at high temperatures [47], directly affecting the final protein concentration.

The fatty acid profile analysis revealed that, after deep-frying, both enriched and non-enriched patties exhibited significant increases in the main fatty acids present in EVOO, particularly palmitic acid (71–93%), OA (73–86%), and α-linolenic acid (ALA; C18:3n3) (100%), compared to other cooking methods. In contrast, LA content was significantly higher in deep-fried non-enriched patties, whereas in enriched patties, it decreased slightly without statistical significance (Table 2). Across all patties and cooking methods, MUFA predominated, followed by SFA and, to a lesser extent, PUFA. OA was the most abundant fatty acid in EVOO-enriched patties and in deep-fried samples, showing significant differences in enriched patties subjected to deep-frying compared to other patties and cooking methods.

The fatty acid profile of raw EVOO showed palmitic acid (7.88 g/100 g oil), OA (59.89 g/100 g oil), and ALA (2.20 g/100 g oil) as its main constituents. After frying, significant modifications were observed in the composition of the oils. The palmitic acid content increased in both used oils (3–8%), particularly in the oil recovered from EVOO-enriched patties, which also exhibited higher levels of ALA compared to the oil from non-enriched parries (*p* ˂ 0.05). Conversely, OA significantly decreased after frying, with raw EVOO showing the highest proportion of OA. Regarding fatty acid classification, the oil used to fry enriched patties showed significantly higher proportions of SFA and PUFA. At the same time, raw EVOO retained the highest MUFA content (82%), reflecting its characteristic composition (Table 3).

The fatty acid composition results are consistent with previous studies [32,48,49]. The addition of EVOO increased MUFA, especially OA, while PUFA decreased during deep-frying. De Alzaa et al. [18] similarly, reported an increase in MUFA and a reduction in PUFA in chicken nuggets and French fries fried in EVOO compared to their raw counterparts (e.g., nuggets: MUFA from 65% to 78%, PUFA from 27% to 8%; French fries: MUFA from 60% to 70%, PUFA from 23% to 11%) [19]. Likewise, broccoli deep-fried in EVOO exhibited a fatty acid profile resembling that of the frying oil (80% MUFA and 4% PUFA) [18]. This pattern has been observed in other foods with low initial lipid content [31]. In the present study, patties fried in EVOO followed a similar trend, with increases in MUFA and SFA, and a decrease in PUFA, particularly LA. This may be related to frying time and the food matrix [48,49]. In non-enriched patties, LA content increased when fried in EVOO, whereas in enriched patties, LA decreased, likely due to PUFA oxidation during frying [45,47,48]. The exchange of fatty acids between food and the frying medium is a critical factor [50]. Higher fatty acid concentrations were observed in the frying oil used for EVOO-enriched patties compared to non-enriched ones, consistent with studies showing that the type of fried food can alter oil composition [48,51]. In this study, EVOO was used for a single frying cycle at 170 °C for 2 min without reuse; however, oil reuse, longer frying times, or higher temperatures could markedly affect fatty acid composition.

From a practical perspective, the available evidence suggests that enriching patties with EVOO and applying cooking methods such as air-frying or baking may offer relevant advantages. Air-frying has been reported to substantially reduce oil uptake (by approximately 70–90% compared with deep-frying), resulting in lower fat contents, reduced lipid degradation, and improved retention of bioactive compounds [29]. Accordingly, EVOO-enriched patties prepared by air-frying or baking may contribute to higher MUFA levels and improved sensory acceptability while limiting overall fat uptake, in line with evidence showing reduced oil absorption and better lipid quality when stable, MUFA-rich oils are used under conditions that minimize oil incorporation [52]. Nevertheless, the present study was limited to acute frying conditions; therefore, future research should evaluate repeated frying cycles to determine whether these trends persist under extended frying scenarios.

From a sustainability perspective, recent evidence suggests that cooking methods such as air-frying and oven baking substantially reduce oil consumption compared to deep-frying, particularly in scenarios involving repeated frying cycles and the reuse of oil. In contrast, single-use, short-time deep-frying conditions, representative of domestic practices, are associated with markedly lower environmental and health-related impacts than prolonged or repeated frying [53]. These findings support the ecological relevance of the frying conditions applied in the present study and highlight the potential advantages of alternative cooking methods in reducing oil use while maintaining product quality.

### 3.2. Total Polyphenol Content and Antioxidant Capacity of Patties with EVOO Prepared Using Different Cooking Methods

**Figure 2:**
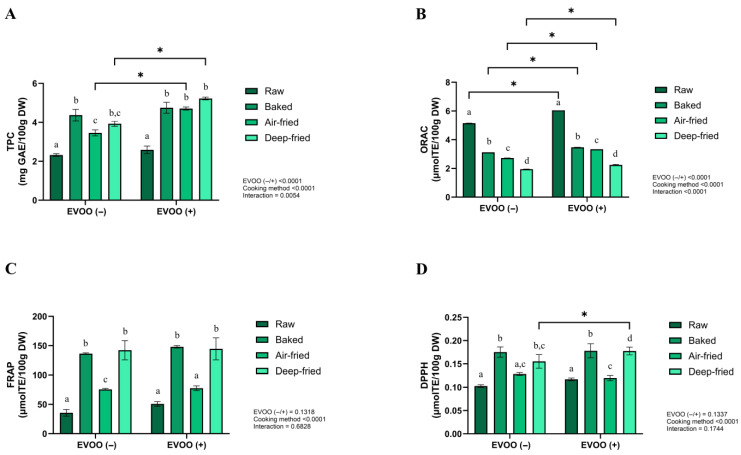
Total polyphenols and antioxidant capacity of vegetable patties with and without added EVOO prepared by different cooking methods. (**A**) Total polyphenol content (mg GAE/100 g DW); (**B**) ORAC (µmol TE/100 g DW). (**C**) FRAP (µmol TE/100 g DW); (**D**) DPPH (µmol TE/100 g DW). Values are mean ± SD. Two-way ANOVA (factors: EVOO addition and cooking method) followed by Tukey’s test. Different lowercase letters above bars denote differences among cooking methods within the same EVOO group (*p* < 0.05). * Indicate differences between EVOO (−) and EVOO (+) within the same cooking method (*p* < 0.05). EVOO, extra-virgin olive oil; GAE, gallic acid equivalents; TE, Trolox equivalents; DW, dry weight.

The impact of cooking method and EVOO enrichment on the total polyphenol content (TPC) and antioxidant capacity of vegetable patties was assessed using widely applied chemical assays (Folin–Ciocalteu, ORAC, FRAP, and DPPH), which are commonly used for evaluating complex food matrices [54]. Although these methods do not aim to replicate physiological conditions and may be influenced by matrix components, they provide valuable information on the relative antioxidant potential of foods subjected to various processing methods [55]. Baked non-enriched patties showed a significant increase in TPC. In contrast, EVOO-enriched patties reached the highest TPC when deep-fried compared to their raw counterparts (*p* < 0.001) (Figure 2A).

Raw patties, regardless of enrichment, displayed the highest ORAC values (*p* < 0.001) (Figure 2B). In the DPPH assay, baked non-enriched patties and deep-fried enriched patties exhibited elevated values, with significant differences only in baked non-enriched patties compared to raw samples (Figure 2C). FRAP values were highest in deep-fried non-enriched patties and baked enriched patties (*p* < 0.05) (Figure 2D). Overall, no significant differences were found in TPC, DPPH, or FRAP across cooking methods, except for ORAC, where raw patties consistently outperformed cooked ones.

**Figure 3:**
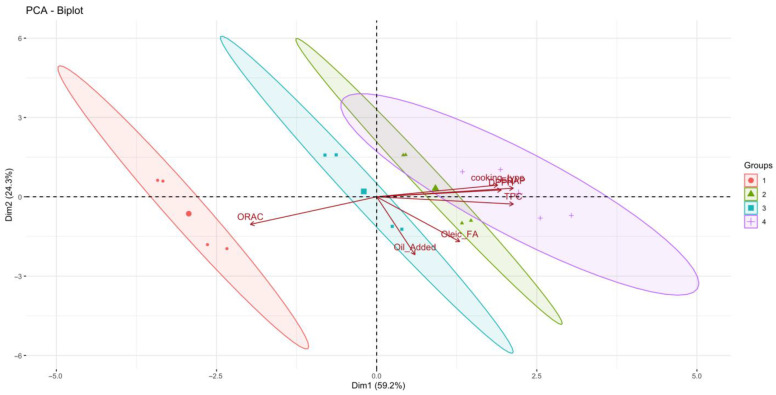
Principal Component Analysis (PCA) biplot showing the projection of samples onto the first two principal components (PC1 and PC2). Vectors represent variable loadings for total polyphenol content (TPC), antioxidant capacity assays (FRAP, DPPH, and ORAC), cooking method, oil addition, and oleic acid content. Sample groups correspond to different cooking methods: 1 = raw, 2 = baked, 3 = air-fried, and 4 = deep-fried. The plot was generated using RStudio with the ggplot2. Own elaboration.

These univariate results were further supported by multivariate analysis. PCA revealed that the first two components explained 83.5% of the total variance, indicating a strong structure within the dataset. PC1, which accounted for 59.2% of the variance, was primarily associated with antioxidant-related variables, including FRAP (loading = 0.4508), TPC (0.4515), and DPPH (0.4110), as well as cooking type (0.3995). Highlighting the central role of thermal processing in modulating antioxidant-related responses. In contrast, ORAC (−0.4167) showed a strong negative loading on PC1, suggesting an inverse. PC2 (24.3% of variance) was mainly driven by EVOO addition (−0.7220) and OA content (−0.5585), separating samples according to lipid enrichment and fatty acid composition rather than antioxidant magnitude (Figure 3).

**Figure 4:**
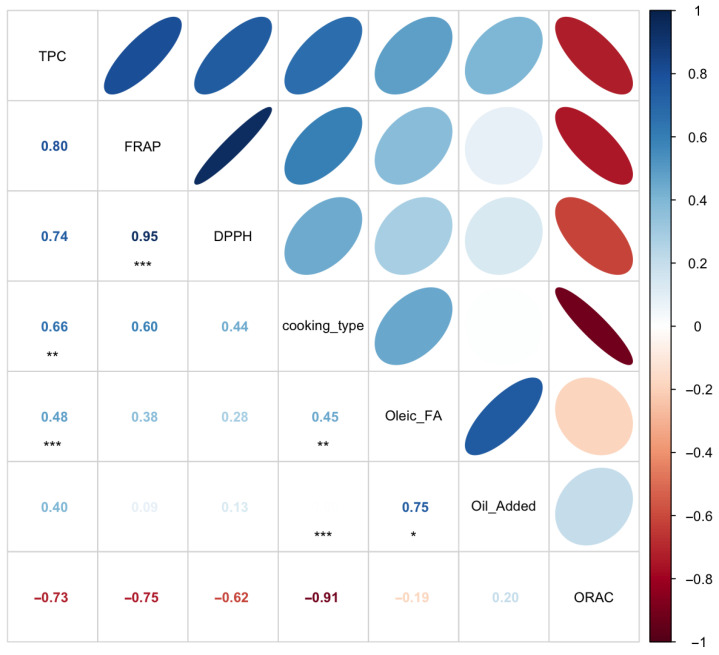
Correlation analysis shown as a correlogram illustrating the relationships among total polyphenol content (TPC), antioxidant capacity assays (FRAP, DPPH, and ORAC), cooking method, oil addition, and oleic acid content. Color intensity and circle size indicate the strength and direction of Pearson correlation coefficients. Statistical significance is indicated as * *p* < 0.05, ** *p* < 0.01, and *** *p* < 0.001. The plot was generated using RStudio with the corrplot package.

Correlation analysis corroborated these findings, revealing strong positive correlations among TPC, FRAP, and DPPH (r = 0.80–0.95), confirming their close association and common response pattern to cooking treatments. Cooking method also showed moderate positive correlations with these antioxidant measures (r = 0.44–0.66), whereas ORAC exhibited strong negative correlations with TPC (−0.73), FRAP (−0.75), DPPH (−0.62), and cooking type (−0.91), reinforcing its distinct response to thermal processing (Figure 4). The strong correlation between EVOO addition and OA content further supports their combined influence on sample differentiation along PC2. Together, these multivariate patterns confirm that antioxidant capacity and cooking conditions primarily drive variability among samples, while EVOO enrichment and lipid composition represent secondary but distinct sources of variation.

The absence of statistically significant differences among treatments should be interpreted within the analytical context of the applied methods. Chemical antioxidant assays, such as the Folin–Ciocalteu, DPPH, FRAP, and ORAC methods, provide comparative information on antioxidant-related responses in complex food matrices. However, their sensitivity may be influenced by matrix components and thermally generated compounds. The combined use of multiple assays, as recommended in previous studies, helps reduce method-specific bias and allows a better comparative interpretation of antioxidant potential [56].

The apparent divergence between ORAC and TPC values can be explained by the different analytical principles underlying these assays. ORAC primarily reflects hydrogen atom transfer (HAT)-based antioxidant activity, which is more sensitive to thermal degradation. In contrast, increases in total polyphenol content, as determined by the Folin–Ciocalteu assay, together with higher values in assays based on single-electron transfer (SET), such as FRAP and DPPH, may be associated with the release and migration of phenolic compounds from EVOO into the food matrix during frying [57].

EVOO’s antioxidant properties are well-documented [58], although its TPC could vary with agronomic and processing factors [59]. In this study, EVOO enrichment increased TPC, particularly with baking, air-frying, or deep-frying, consistent with previous findings [18,60]. Moreover, it has been reported that foods with higher moisture content tend to absorb more oil, facilitating antioxidant transfer to the final product [59,61]. During deep-frying, post-frying cooling can further increase TPC due to the migration of antioxidant compounds from the EVOO [62]. The vegetable matrix also contributed, with ingredients such as onions, garlic, broccoli, and carrots providing additional phenolics [63]. Cooking could promote the release of bound phenols through cell wall softening and fiber breakdown [63,64,65]. In addition, the cleavage of phenol–sugar glycosidic bonds, resulting in aglycones, could also contribute to increased phenolic concentration [64,65]. This effect has been observed in baked, microwaved, and boiled vegetables, including leafy greens and broccoli [18,64,65]. However, excessive heating may reduce TPC and antioxidant capacity via oxidation and polymerization reactions, hydrolysis, and the formation of covalent bonds between oxidized phenols and proteins or amino acids [17,19,46]. These findings highlight the importance of cooking conditions in preserving antioxidant compounds.

EVOO is recognized for its high ORAC values [19,66], linked to phenolics, carotenoids, and vitamins C and E, with possible synergistic effects [67,68]. Deep-frying reduced ORAC in some cases due to thermal degradation of phenolics, as reported by Xu et al. [69] for broccoli. In the DPPH assay, most patties maintained or improved their antioxidant capacity, regardless of the cooking method, which was influenced by the phenol structure and assay conditions [19,49,60]. The structural characteristics of phenolic compounds, such as the presence of glycosidic residues and the position of free and esterified hydroxyl groups, play a key role in their reactivity toward the DPPH radical [67]. On the other hand, FRAP values increased after thermal treatments, particularly after deep-frying, likely due to the formation of Maillard reaction products with antioxidant activity [19,70].

Despite these findings, it is worth noting that the present study focused on antioxidant capacity assessed through chemical assays and did not include specific markers of frying-related oil degradation, such as total polar compounds, *p*-anisidine value, or volatile oxidation products. While the deep-frying conditions applied (single use of fresh EVOO, short frying time, and moderate temperature) are representative of domestic cooking practices, previous studies have demonstrated that the formation of oxidation and degradation products in EVOO is mainly associated with prolonged and repeated frying cycles [71]. Accordingly, under the short and controlled frying conditions used in this study, the formation of these compounds is expected to be limited, although not directly assessed. Nevertheless, their evaluation would allow a more comprehensive assessment of quality and safety aspects. Future studies should therefore incorporate these parameters, particularly under extended frying times or conditions of oil reuse.

### 3.3. Sensory Evaluation

A sensory evaluation with 53 participants assessed the acceptability of vegetable patties cooked by baking, air-frying, or deep-frying in EVOO. Most participants were women (77%), aged 18–20 years (40%), all of whom consumed the ingredients used in the patties and reported no allergies. Participants were recruited mainly from university students and staff. Despite the predominance of women and young adults, comparable demographic compositions have been frequently reported in consumer evaluations of patties. For example, a sensory evaluation of low-sodium patties recruited 51 untrained college students. The panel consisted of 39 women and 12 men (approximately 76% female), and participants (aged 19–23 years) evaluated attributes using a seven-point hedonic scale [72]. Likewise, a study assessing beef-patty analogues recruited 118 panelists (70 women and 48 men) drawn from university staff and students, with 36.4% of the participants falling within the 18–24-year age range. This study reported that younger consumers often exhibit distinct flavor preferences and health-related expectations [73]. These antecedents suggest that a predominantly female, university-based panel can still provide meaningful insights into the target demographic for healthier patties; however, this distribution may limit the generalizability of the results to broader consumer populations.

**Figure 5:**
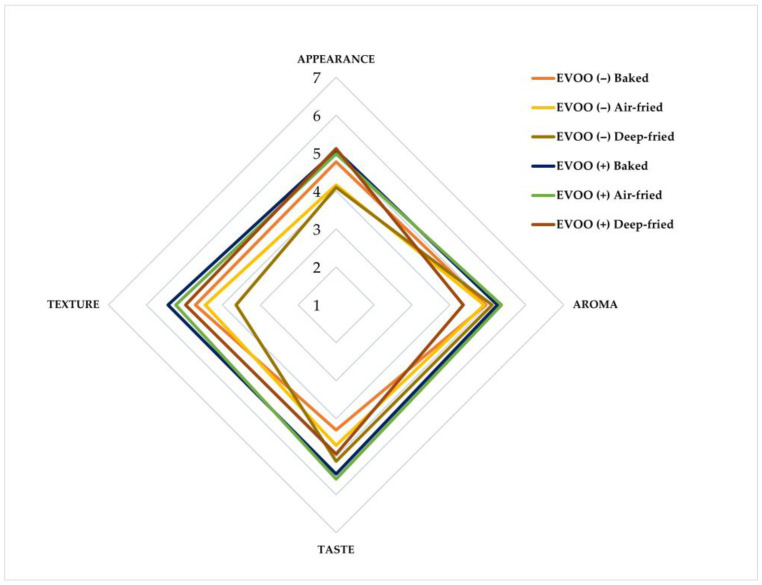
Sensory profiles of vegetable patties formulated with or without extra-virgin olive oil (EVOO) and subjected to baking, air-frying, or deep-frying, represented as a radar (net-type) plot. Mean scores for appearance, aroma, taste, and texture were obtained from a consumer sensory evaluation using a 7-point hedonic scale, 1 = dislike very much, 7 = like very much. EVOO, extra-virgin olive oil; EVOO (−), vegetable patties formulated without EVOO; EVOO (+), vegetable patties formulated with EVOO.

Baked patties and air-fried patties with EVOO achieved the highest ratings across all attributes (Figure 5). For baked patties, aroma, taste, and texture scored significantly higher (*p* < 0.05), while air-fried patties showed significant improvements in all attributes. Deep-fried patties showed mixed results: oil-free formulations were preferred for aroma (*p* < 0.05) and taste, whereas EVOO-enriched patties scored higher in appearance and texture (*p* < 0.05) (Table 4). Overall, consumer preference favored EVOO-enriched formulations, with 77% of participants preferring baked patties with EVOO and 89% preferring air-fried patties with EVOO, whereas preference for deep-fried patties was more evenly distributed between EVOO-enriched and non-enriched formulations (53% vs. 47%).

Sensory differences were primarily influenced by the cooking method and the use of EVOO enrichment. It has been reported that the appearance of fried food could be affected by color, opacity, and heterogeneity [74]. During concentration cooking methods, foods undergo Maillard reactions or caramelization, leading to nutrient loss and product darkening, as corroborated by previous studies [13,69,74]. The intensity of browning in EVOO-enriched patties is linked to lysine, histidine, and methionine loss [18]. In this study, EVOO formulations received the highest appearance scores, likely due to their color and opacity, which strongly influence visual perception [74]. Aroma and taste ratings were higher for baked and air-fried EVOO patties, while deep-fried non-enriched patties were also preferred. These attributes are shaped by lipid oxidation, Maillard reactions, and amino acid degradation, producing volatile compounds that enhance taste [17,74] which makes them more appetizing and preferred [60,75]. Moreover, the distinctive fruity taste and aroma of EVOO enhanced the appeal of the enriched patties [58]. Texture scores correlated negatively with moisture content, with non-enriched patties rated lower due to higher moisture [13]. Similar findings were reported by De Alzaa et al. [18], who found a higher taste preference for deep-fried potatoes in EVOO compared to canola oil.

Food appearance is influenced by oil type, temperature, frying time, and sample size, and is closely linked to consumer perception of quality [49]. In this study, the panel consisted mainly of young volunteers rather than trained assessors, which may have introduced bias linked to their dietary habits [60]. EVOO is also less commonly consumed in Chile compared to Mediterranean countries [19]. Moreover, the patty formulation, based on oat flour rather than conventional wheat flour or starches, may have influenced sensory responses [74]. Despite some significant attribute differences, overall ratings across cooking methods were similar, suggesting that broader demographic representation in future panels could provide a more comprehensive understanding of consumer preferences.

## 4. Conclusions

This study demonstrates that cooking method markedly influences nutritional composition, fatty acid profile, antioxidant capacity, and sensory acceptability of vegetable patties formulated with and without EVOO. Deep-frying substantially increased the fat content, whereas baking and air-frying limited fat absorption, offering healthier alternatives. EVOO enrichment promoted the incorporation of MUFA, particularly OA, and reduced PUFA, while facilitating the transfer of polyphenolic compounds to the patties.

These effects, combined with the antioxidant contribution of vegetable ingredients, improved the lipid profile and enhanced antioxidant quality, especially when using less aggressive cooking methods. In terms of sensory performance, EVOO-enriched patties cooked by air-frying achieved the highest overall acceptance, followed by baked patties, with improvements in aroma, taste, and texture that aligned with consumer preferences. Overall, incorporating EVOO into vegetable patty formulations, combined with baking or air-frying, emerges as a practical strategy for producing nutritionally improved, sensorially appealing, and more sustainable products that meet current demands for healthier food alternatives. Future studies should aim to address some of the limitations of the present work by exploring the effect of multiple frying cycles, using more representative panels for sensory analysis, along with specific chemical analyses of oil oxidation and biological approaches, to further validate and extend the observed trends.

## Figures and Tables

**Table 1:** Proximate composition of vegetable patties with and without extra-virgin olive oil prepared by four cooking methods.

	EVOO (−)				EVOO (+)			
	Raw	Baked	Air-Fried	Deep-Fried	Raw	Baked	Air-Fried	Deep-Fried
Moisture (%)	76.13 ± 0.41 ^a^	73.69 ± 0.29 ^b^	71.31 ± 0.44 ^c^	62.26 ± 0.59 ^d^	71.18 ± 0.39 ^a,^*	70.09 ± 0.22 ^b,^*	68.58 ± 0.41 ^c,^*	59.03 ± 0.32 ^d,^*
Water activity (A_W_)	0.88 ± 0.03 ^a^	0.9 ± 0.01 ^a^	0.89 ± 0.01 ^a^	0.97 ± 0.01 ^b^	0.91 ± 0.02 ^a,^*	0.92 ± 0.01 ^a^	0.89 ± 0.02 ^a^	0.96 ± 0.01 ^b^
Ash (% DW)	5.76 ± 0.02 ^a^	6.72 ± 0.01 ^b^	7.13 ± 0.16 ^c^	4.18 ± 0.02 ^d^	4.43 ± 0.02 ^a,^*	5.42 ± 0.01 ^b,^*	5.51 ± 0.04 ^b,^*	3.46 ± 0.01 ^c,^*
Protein (% DW)	21.69 ± 0.22 ^a^	22.89 ± 0.66 ^a^	22.0 ± 0.23 ^a^	15.71 ± 0.43 ^b^	17.99 ± 0.74 ^a,^*	18.88 ± 0.20 ^a,^*	18.23 ± 0.64 ^a,^*	12.68 ± 0.16 ^b,^*
Total fat (% DW)	5.58 ± 0.34 ^a^	6.02 ± 0.03 ^a,b^	6.20 ± 0.05 ^b^	22.41 ± 0.33 ^c^	16.98 ± 0.32 ^a,^*	18.74 ± 0.67 ^b,^*	12.38 ± 0.24 ^c,^*	30.88 ± 0.6 ^d,^*
Total carbohydrates (% DW)	66.97 ± 0.54 ^a^	64.37 ± 0.7 ^b^	64.67 ± 0.33 ^b^	57.41 ± 0.33 ^c^	60.59 ± 0.44 ^a,^*	56.96 ± 0.46 ^b,^*	63.88 ± 0.35 ^c^	52.98 ± 0.75 ^d,^*

Data are expressed as mean ± standard deviation (g/100 g DW). Two-way ANOVA followed by Tukey’s multiple comparison test was applied. Different lowercase superscripts (a–d) indicate significant differences between cooking methods within the same group (without EVOO (−) or with added EVOO (+)). * Denotes significant differences between patties with and without EVOO prepared using the same cooking method (*p* < 0.05). EVOO: extra-virgin olive oil; DW: dry weight; A_W_: water activity.

**Table 2:** Fatty acid composition (g/100 g DW) of vegetable patties with and without extra-virgin olive oil prepared by four cooking methods.

	EVOO (−)				EVOO (+)			
	Raw	Baked	Air-Fried	Deep-Fried	Raw	Baked	Air-Fried	Deep-Fried
C14:0 (myristic)	0.01 ± 0.00 ^a^	0.01 ± 0.00 ^a^	0.01 ± 0.00 ^a^	0.01 ± 0.00 ^a^	0.01 ± 0.00 ^a^	0.00 ± 0.00 ^b^	0.01 ± 0.00 ^a^	0.01 ± 0.00 ^a^
C16:0 (palmitic)	1.05 ± 0.01 ^a^	0.87 ± 0.03 ^b^	0.91 ± 0.01 ^b^	3.58 ± 0.07 ^c^	1.84 ± 0.06 ^a,^*	1.72 ± 0.05 ^b,^*	1.72 ± 0.01 ^b,^*	5.80 ± 0.03 ^c,^*
C16:1 (palmitoleic)	0.01 ± 0.00 ^a^	0.01 ± 0.00 ^a^	0.02 ± 0.00 ^b^	0.15 ± 0.01 ^c^	0.07 ± 0.00 ^a,^*	0.06 ± 0.00 ^b,^*	0.07 ± 0.00 ^a,^*	0.24 ± 0.00 ^c,^*
C18:0 (stearic)	0.16 ± 0.00 ^a^	0.13 ± 0.00 ^b^	0.13 ± 0.00 ^b^	0.74 ± 0.02 ^c^	0.39 ± 0.01 ^a,^*	0.35 ± 0.01 ^b,^*	0.36 ± 0.00 ^b,^*	1.26 ± 0.02 ^c,^*
C18:1n9c (oleic)	2.97 ± 0.06 ^a^	2.42 ± 0.07 ^b^	2.52 ± 0.01 ^b^	20.65 ± 0.02 ^c^	10.01 ± 0.00 ^a,^*	9.37 ± 0.05 ^b,^*	9.91 ± 0.49 ^a,^*	37.15 ± 0.08 ^c,^*
C18:2n6c (linoleic)	2.37 ± 0.00 ^a,b^	2.18 ± 0.06 ^b^	2.13 ± 0.01 ^b^	2.53 ± 0.03 ^a^	1.92 ± 0.38 ^a,^*	1.57 ± 0.03 ^b,^*	1.45 ± 0.09 ^b,^*	1.49 ± 0.03 ^b,^*
C18:3n6 (γ-linolenic)	0.06 ± 0.00 ^a^	0.05 ± 0.00 ^b^	0.05 ± 0.00 ^b^	0.12 ± 0.00 ^c^	0.07 ± 0.00 ^a,^*	0.06 ± 0.00 ^b,^*	0.06 ± 0.00 ^b,^*	0.18 ± 0.01 ^c,^*
C18:3n3 (α-linolenic)	0.00 ± 0.00 ^a^	0.00 ± 0.00 ^a^	0.00 ± 0.00 ^a^	0.15 ± 0.00 ^b^	0.00 ± 0.00 ^a^	0.00 ± 0.00 ^a^	0.01 ± 0.00 ^b,^*	0.27 ± 0.00 ^c,^*
Total SFA	1.22 ± 0.01 ^a^	1.00 ± 0.03 ^b^	1.05 ± 0.01 ^b^	4.33 ± 0.09 ^c^	2.23 ± 0.05 ^a,^*	2.07 ± 0.06 ^b,^*	2.08 ± 0.00 ^b,^*	7.07 ± 0.01 ^c,^*
Total MUFA	2.98 ± 0.06 ^a^	2.43 ± 0.07 ^b^	2.54 ± 0.01 ^a,b^	20.80 ± 0.03 ^c^	10.07 ± 0.00 ^a,^*	9.44 ± 0.05 ^b,^*	9.98 ± 0.49 ^b,^*	37.40 ± 0.08 ^c,^*
Total PUFA	2.44 ± 0.00 ^a^	2.23 ± 0.06 ^a^	2.19 ± 0.01 ^a^	2.80 ± 0.03 ^b^	1.99 ± 0.38 ^a,^*	1.63 ± 0.03 ^b,c,^*	1.51 ± 0.09 ^b,^*	1.94 ± 0.03 ^c,^*
Total FA	6.63 ± 0.07 ^a^	5.66 ± 0.04 ^b^	5.78 ± 0.03 ^b^	27.93 ± 0.09 ^c^	14.30 ± 0.43 ^a,^*	13.14 ± 0.07 ^b,^*	13.57 ± 0.41 ^b,^*	46.41 ± 0.12 ^c,^*

Data are expressed as mean ± standard deviation (g/100 g DW). Two-way ANOVA followed by Tukey’s multiple comparison test was applied. Different lowercase superscripts (a, b, c) indicate significant differences between cooking methods within the same group (without EVOO (−) or with added EVOO (+)). * Denotes significant differences between patties with and without EVOO prepared using the same cooking method (*p* < 0.05). EVOO: Extra-virgin olive oil; SFA: Saturated fatty acids; MUFA: Monounsaturated fatty acids; PUFA: Polyunsaturated fatty acids; FA: Fatty acids.

**Table 3:** Fatty acid profile (g/100 g oil) of extra-virgin olive oil before frying and of used frying oils after deep-frying vegetable patties without (EVOO−) or with added EVOO (EVOO+).

	Pre-Fry EVOO (Fresh Oil)	Used Oil After Frying EVOO (−) Patty	Used Oil After Frying EVOO (+) Patty
C14:0 (myristic)	0.05 ± 0.01	0.06 ± 0.02	0.06 ± 0.00
C16:0 (palmitic)	7.88 ± 0.12 ^a^	8.13 ± 0.05 ^b^	8.58 ± 0.05 ^c^
C16:1 (palmitoleic)	0.38 ± 0.01	0.36 ± 0.01 ^a^	0.39 ± 0.00 ^a^
C18:0 (stearic)	1.82 ± 0.06	1.77 ± 0.04	1.86 ± 0.04
C18:1n9c (oleic)	59.89 ± 0.13 ^a^	56.33 ± 0.04 ^b^	58.36 ± 0.01 ^c^
C18:2n6c (linoleic)	1.00 ± 0.04 ^a^	1.16 ± 0.03	1.18 ± 0.07
C18:3n6 (γ-linolenic)	0.32 ± 0.01	0.32 ± 0.01	0.33 ± 0.01
C18:3n3 (α-linolenic)	2.20 ± 0.01 ^a^	2.09 ± 0.02 ^b^	2.40 ± 0.06 ^c^
Total SFA	9.75 ± 0.17	9.97 ± 0.12	10.50 ± 0.01 ^a^
Total MUFA	60.27 ± 0.11 ^a^	56.70 ± 0.05 ^b^	58.75 ± 0.01 ^c^
Total PUFA	3.52 ± 0.01	3.57 ± 0.00	3.91 ± 0.13 ^a^
Total FA	73.55 ± 0.04 ^a^	70.23 ± 0.17 ^b^	73.16 ± 0.12 ^c^

Data means SD (g/100 g oil). One-way ANOVA followed by Tukey’s test. Different lowercase letters indicate significant differences between oils (*p* < 0.05). EVOO: extra-virgin olive oil. EVOO (−): patty without added oil. EVOO (+): patty with added EVOO. SFA: saturated fatty acids; MUFA: monounsaturated fatty acids; PUFA: polyunsaturated fatty acids; FA: fatty acids.

**Table 4:** Sensory attributes of vegetable patties with and without added EVOO prepared by three cooking methods.

	EVOO (−)			EVOO (+)		
Sensory Attribute	Baked	Air-Fried	Deep-Fried	Baked	Air-Fried	Deep-Fried
Appearance	4.77 ± 1.37 ^a^	4.16 ± 1.35 ^a,b^	4.10 ± 1.59 ^b^	5.07 ± 1.12 ^a^	4.98 ± 1.33 ^a,^*	5.12 ± 1.23 ^a,^*
Aroma	4.94 ± 1.09 ^a^	4.90 ± 1.13 ^a^	5.11 ± 1.17 ^a^	5.25 ± 1.03 ^a^	5.36 ± 0.91 ^a,^*	4.35 ± 1.65 ^b,^*
Taste	4.30 ± 1.41 ^a^	4.70 ± 1.23 ^a,b^	5.12 ± 1.42 ^b^	5.46 ± 1.00 ^a,b,^*	5.59 ± 1.02 ^a,^*	4.93 ± 1.39 ^b^
Texture	4.71 ± 1.36 ^a^	4.45 ± 1.57 ^a^	3.64 ± 1.88 ^b^	5.43 ± 0.95 ^a,^*	5.23 ± 1.25 ^a,^*	4.97 ± 1.37 ^a,^*

The data are presented as mean ± standard deviation. Two-way ANOVA followed by Tukey’s multiple comparison test was applied. Different lowercase superscripts (a, b) indicate significant differences between cooking methods within the same group (without EVOO (−) or with added EVOO (+)). * Denotes significant differences between patties with and without EVOO prepared using the same cooking method (*p* < 0.05). EVOO: Extra-virgin olive oil.

## Data Availability

The original contributions presented in this study are included in the article. Further inquiries can be directed to the corresponding authors.
